# Identification and Analysis of Necroptosis-Related Genes in COPD by Bioinformatics and Experimental Verification

**DOI:** 10.3390/biom13030482

**Published:** 2023-03-06

**Authors:** Yingxi Wang, Xin Su, Yan Yin, Qiuyue Wang

**Affiliations:** 1 Institute of Respiratory Disease, Department of Pulmonary and Critical Care Medicine, The First Hospital of China Medical University, Shenyang 110001, China; 2 Department of Plastic Surgery, The First Hospital of China Medical University, Shenyang 110001, China

**Keywords:** necroptosis, chronic obstructive pulmonary disease, immune infiltration, diagnostic biomarker, TF–miRNA coregulatory network, potential therapeutic drug

## Abstract

Chronic obstructive pulmonary disease (COPD) is a heterogeneous and complex progressive inflammatory disease. Necroptosis is a newly identified type of programmed cell death. However, the role of necroptosis in COPD is unclear. This study aimed to identify necroptosis-related genes in COPD and explore the roles of necroptosis and immune infiltration through bioinformatics. The analysis identified 49 differentially expressed necroptosis-related genes that were primarily engaged in inflammatory immune response pathways. The infiltration of CD8+ T cells and M2 macrophages in COPD lung tissue was relatively reduced, whereas that of M0 macrophages was increased. We identified 10 necroptosis-related hub genes significantly associated with infiltrated immune cells. Furthermore, 7 hub genes, CASP8, IL1B, RIPK1, MLKL, XIAP, TNFRSF1A, and CFLAR, were validated using an external dataset and experimental mice. CFLAR was considered to have the best COPD-diagnosing capability. TF and miRNA interactions with common hub genes were identified. Several related potentially therapeutic molecules for COPD were also identified. The present findings suggest that necroptosis occurs in COPD pathogenesis and is correlated with immune cell infiltration, which indicates that necroptosis may participate in the development of COPD by interacting with the immune response.

## 1. Introduction

Chronic obstructive pulmonary disease (COPD) is a progressive inflammatory disease of the airways, alveoli, and microvessels characterized by persistent respiratory symptoms and incompletely reversible airflow limitation [1,2]. Tobacco smoking and exposure to indoor air pollution (including biomass combustion), ambient air pollution, and occupational pollutants have been reported as leading risk factors in most settings [3]. The main pulmonary pathologies of COPD include chronic bronchitis, airway remodeling, and emphysema. COPD is the third most common cause of death worldwide [4]. Still, the current treatments are limited to controlling symptoms and reducing exacerbations and exhibit an inability to repair defective tissues and modify the course of the disease. Therefore, an understanding of the pathogenesis of COPD is imperative for guiding clinical diagnosis and treatment and achieving improved clinical efficacy.

Necroptosis is a newly recognized genetically regulated form of necrotic cell death that integrates some features of necrosis and apoptosis [5,6]. The necroptosis pathway is induced by a variety of intracellular signals and regulated by receptor-interacting protein kinases 1 and 3 (RIPK1 and RIPK3) and mixed-lineage kinase domain-like pseudokinase (MLKL), which form a regulatory necrosome complex [5,7,8]. Phosphorylated MLKL facilitates the formation of membrane-disrupting pores, ultimately leading to necrotic death and the release of proinflammatory intracellular contents. Accumulating evidence has implicated necroptosis in the pathogenesis of immune system disorders, inflammatory diseases, and cancer [9,10,11,12]. Some findings have indicated the roles of necroptosis and its regulatory proteins in COPD [13,14,15,16]. For example, necroptosis is induced in human COPD and mice after smoke exposure, and genetic or pharmacologic inhibition can attenuate cigarette smoke (CS)-induced airway inflammation, airway remodeling, and emphysema. However, the necroptosis-related genes (NRGs) in COPD remain largely unknown and need to be further explored.

**Figure 1:**
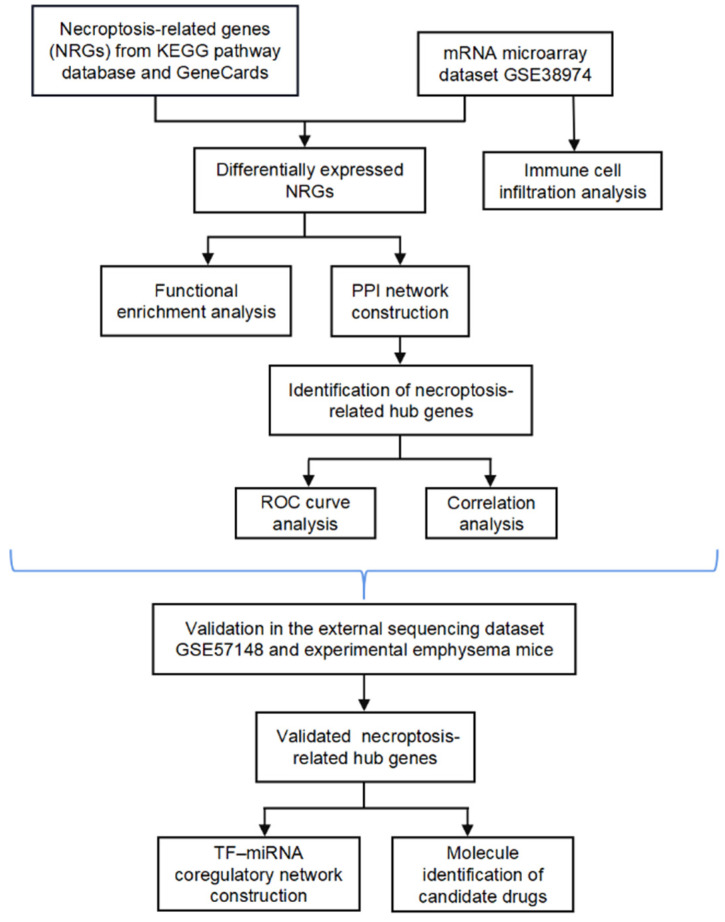
Flow chart.

In the present study, we systematically analyzed the differential expression profiles of NRGs between normal and COPD tissues using the microarray dataset GSE38974. Moreover, the potential functional mechanism and hub genes associated with necroptosis were explored, and the relationship between necroptosis and infiltrating immune cells was examined. We further externally verified the hub NRGs using another sequencing dataset (GSE57148) and experimental animals and evaluated the diagnostic value of the hub genes. We also constructed a transcription factor (TF)–miRNA coregulatory network for the verified NRGs and identified candidate drug molecules. The bioinformatics analysis of this study was conducted according to Figure 1.

## 2. Materials and Methods

### 2.1. Selection of NRGs

A profile of 159 human necroptosis genes was collected from the Kyoto Encyclopedia of Genes and Genomes (KEGG) pathway database (https://www.kegg.jp/kegg/pathway.html, accessed on 11 June 2022) [17]. Additionally, 99 genes associated with necroptosis were acquired from GeneCards (https://www.genecards.org/, accessed on 11 June 2022) based on a relevance score > 1.0 [18]. The two gene profiles were combined to obtain 232 NRGs. Table S1 in the Supplementary Materials shows further details.

### 2.2. Data Acquisition

The normalized expression matrix of the microarray dataset GSE38974 contained 23 COPD and 9 normal lung tissue samples and was obtained with the GPL4133 platform (Agilent-014850 Whole Human Genome Microarray 4 × 44K G4112F). This dataset was used to screen differentially expressed NRGs. The gene expression levels in the high-throughput sequencing dataset GSE57148, which included 98 COPD and 91 normal lung tissue samples and was based on the GPL11154 platform (Illumina HiSeq 20009, *Homo sapiens*), were normalized using transcripts per million (TPM). The PCA plot was generated using the “ggplot2” package of R software (version 4.2.1) [19]. All data used in the study are publicly accessible in the GEO database (http://www.ncbi.nlm.nih.gov/geo/, accessed on 9 June 2022) [20]. Detailed information on the datasets is shown in Table 1.

### 2.3. Differentially Expressed NRGs

The package “limma” of R software was used to identify differentially expressed NRGs [21], which met the following criteria: false discovery rate < 0.05 and |log2 (fold-change)| > 0.58. Volcano plots, heatmaps, and box plots were generated with the “ggplot2” and “pheatmap” packages [19].

### 2.4. Functional Enrichment Analysis

Gene Ontology (GO) functional enrichment analysis, which included the biological process (BP), cellular component (CC), and molecular function (MF) categories, and KEGG pathway analysis were performed with the R packages “clusterProfiler” and “GOplot” [19,22]. The package “org.Hs.eg.db” was applied to convert the probe IDs [23]. The Z scores were calculated using the “GOplot” package by integrating the expression levels [24]. A Z score greater than 0 indicates positive regulation, and a Z score lower than zero indicates negative regulation.

### 2.5. Protein–Protein Interaction (PPI) Analysis and Hub Gene Identification

The online STRING database (https://string-db.org/, accessed on 11 June 2022) and Cytoscape software (version 3.8.1) were used to analyze the interactions among the differentially expressed NRGs [25,26]. The top 10 potential hub genes were identified based on the cytoHubba degree algorithm [27]. The plugin MCODE was used for cluster analysis of the PPI network [28]. We combined the top 10 potential hub genes and the genes involved in the most significant module to identify the overlapping genes, which were ultimately regarded as hub genes related to necroptosis. The correlations of the hub genes were analyzed using the Spearman correlation in the “corrplot” package, and differences with *p* < 0.05 were considered statistically significant [29].

### 2.6. Evaluation of Immune Cell Infiltration

CIBERSORT is widely used to calculate the abundance of immune cells in the microenvironment [30]. The LM22 signature was downloaded from the CIBERSORTx website (https://cibersortx.stanford.edu/, accessed on 11 June 2022). In this study, we utilized CIBERSORT-based deconvolution combined with LM22 to measure the relative proportion of 22 types of immune subpopulations in lung samples in GSE38974. The infiltrating immune cell composition in each sample was visualized using the “ggplot” package, and 22 immune cell subtypes in the COPD and normal groups were compared using the packages “ggpubr” and “cowplot” [31]. The “corrplot” package was used to analyze the correlations among differentially infiltrated immune cells. A Spearman correlation analysis of the hub genes and infiltrating immune cells was performed using the “ggstatsplot” package [32].

### 2.7. Validation of Necroptosis-Related Hub Genes in Other Datasets

The expression levels of the necroptosis-related hub genes were extracted from the independent external validation dataset GSE57148, and the difference between COPD and normal lung tissues was calculated and visualized with the packages “ggpurb” and “ggplot2”, with *p* < 0.05 considered to indicate statistical significance.

### 2.8. Animal Model of Cigarette Smoke (CS)-Induced Emphysema

Twelve male C57BL/6J mice (8–10 weeks, 18–20 g) were purchased from Changsheng Biotechnology Company (Liaoning, China). The mice were randomly divided into 2 groups: (1) the normal control group (*n* = 6), and (2) the CS group (*n* = 6). Based on previous studies [33,34], the mice in the CS group underwent whole-body exposure to smoke from 20 Marlboro cigarettes (Philip Morris Companies, 0.8 mg of nicotine, 10 mg of CO, and 10 mg of tar per cigarette) for 40 min in a HOPE-MED 8050 inhalation exposure system (HOPE company, Tianjin, China) twice a day and 6 days per week, whereas the mice in the normal control group were exposed to normal air. After 12 weeks, immediately following sacrifice, the left lung was inflated with 10% paraformaldehyde at a constant pressure of 25 cm H_2_O, and the right lung tissues were removed and stored at −80 °C. The fixed lungs were embedded in paraffin. The design and protocol of the animal experiments were approved by the Animal Care and Use Committee of the China Medical University.

### 2.9. Lung Morphometric Analysis

Paraffin sections (4 μm) were stained with hematoxylin and eosin (H&E) according to conventional protocols. The morphology of the lung tissues was assessed with respect to emphysema changes based on the mean linear intercept (MLI) and mean alveolar number (MAN) at 100× magnification, as previously described [35,36].

### 2.10. Cell Death Assessment

Terminal deoxynucleotidyl transferase-mediated dUTP nick end-labeling (TUNEL) was performed with a TUNEL Assay Kit-HRP-DAB (Abcam, Cambridge, UK) following the manufacturer’s protocol. TUNEL staining can detect both apoptosis and necrosis, including necroptosis [37]. The ratio of TUNEL-positive cells to total cells was measured in a population of more than 3000 parenchymal cells of each lung sample in each group.

### 2.11. Immunohistochemical Staining

Immunohistochemical staining was conducted according to the manufacturer’s instructions. The primary antibodies were as follows: rabbit anti-MLKL (1:2000 dilution, Biorbyt, Cambridge, UK), rabbit polyclonal anti-RIPK1 (1:1000 dilution, Abcam, Cambridge, UK), and rabbit polyclonal anti-RIPK3 (1:500 dilution, Abcam, Cambridge, UK). The mean optical density of positive cells was determined using ImageJ software (version 1.53e) based on the optical density of stained positive cells normalized to the total area of cells in each view at 400× magnification.

### 2.12. Quantitative Reverse Transcription Polymerase Chain Reaction

Gene expression analysis was performed as previously described [38]. Total RNA was extracted from lung tissue using RNAiso Plus (Takara, Kusatsu, Japan). An Evo M-MLV RT Kit with gDNA Clean for qPCR II (Accurate Biotech, Changsha, China) was used to remove the mixed genomic DNA from RNA, and complementary DNA (cDNA) was obtained by reverse transcription of the mRNA. The cDNA was subjected to a real-time quantitative polymerase chain reaction (PCR) with a Roche 480 LightCycler (Roche, Basel, Switzerland) and an SYBR Green Premix Pro Taq HS qPCR Kit (Accurate Biotech, Changsha, China). According to the manufacturer’s instructions, the PCR settings were as follows: initial denaturation for 30 s at 95 °C, followed by 40 cycles of 5 s at 95 °C and 40 s at 59 °C. Relative quantification was performed with the 2^−ΔΔCt^ method based on endogenous control (GAPDH). The primers were synthesized by Takara Biotechnology Company (Dalian, China); Table S2 in the Supplementary Materials details all the primers used in this study.

### 2.13. Receiver Operating Characteristic (ROC) Curve Analysis

ROC curve analysis was performed using the “pROC” and “ggplot2” packages [39]. The area under the ROC curve (AUC) was calculated to evaluate the diagnostic efficacy of the necroptosis-related hub genes. The AUC combines sensitivity and specificity to validate the intrinsic efficacy of diagnostic markers [40]. If the AUC is greater than 0.5, the closer the AUC is to 1, the better the diagnostic effect is. In our study, an AUC greater than 0.7 was considered the ideal diagnostic value.

### 2.14. Construction of a TF–miRNA Coregulatory Network

The RegNetwork repository (https://regnetworkweb.org/, accessed on 14 July 2022) aids the detection of miRNAs and regulatory TFs that regulate differentially expressed genes of interest at the posttranscriptional and transcriptional levels [41]. We collected TF–miRNA coregulatory interactions from this repository using the validated necroptosis-related hub genes. A TF–miRNA coregulatory network was then visualized using NetworkAnalyst (https://www.networkanalyst.ca/, accessed on 14 July 2022), which helps researchers easily navigate complex datasets to identify biological features and functions and thus reach an effective biological hypothesis [42]. The TF–miRNA coregulatory network reflected the miRNA and TF interactions with common hub gene targets and may thus help explain the regulation of the expression of NRGs.

### 2.15. Potential Therapeutic Drug Prediction

The DSigDB database in Enrichr (https://maayanlab.cloud/Enrichr/, accessed on 14 July 2022) is a drug prediction database that can be used to select candidate drugs that potentially target certain genes [43]. These drugs may be therapeutic agents for COPD that act by modulating necroptosis. The PubChem database (https://pubchem.ncbi.nlm.nih.gov/, accessed on 14 July 2022) was used to retrieve the molecular structures of the drugs [44].

### 2.16. Statistical Analysis

R software was used to calculate the significance of the differential expression of NRGs by the Wilcoxon rank sum test. Gene expression in experimental animal samples was statistically analyzed with the Student’s *t*-test using GraphPad Prism (version 8.0.1, GraphPad Software). The results were depicted as the means ± SEMs, and *p* < 0.05 was considered to indicate significance.

## 3. Results

### 3.1. Differential Expression of NRGs

**Figure 2:**
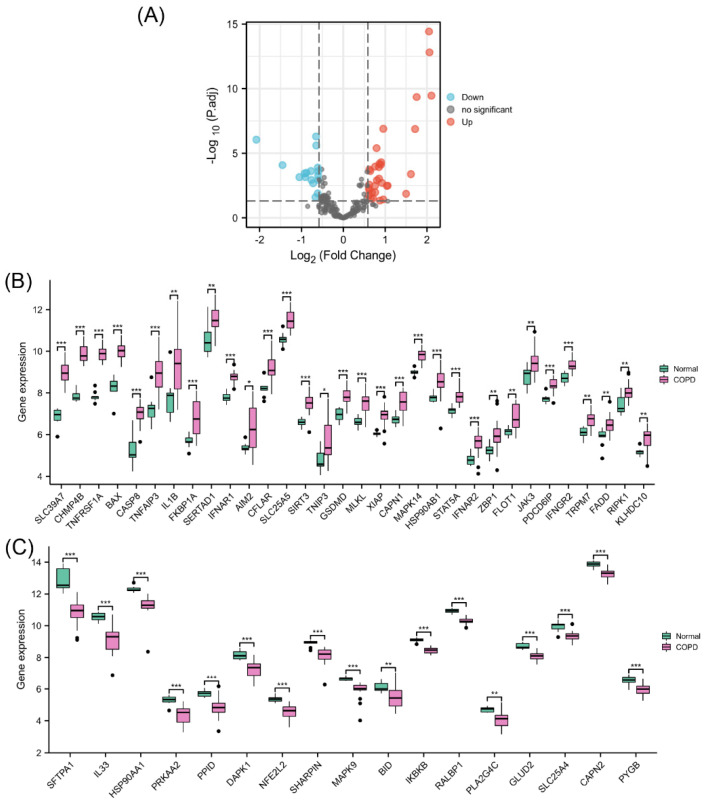
Identification of differentially expressed NRGs. (**A**) Volcano plot of NRGs showing differential expression between the COPD and normal groups. (**B**) Box plot of 32 upregulated differentially expressed NRGs. (**C**) Box plot of 17 downregulated differentially expressed NRGs. * *p* < 0.05, ** *p* < 0.01, *** *p* < 0.001.

The PCA results showed a good clustering degree between the two groups in GSE38974 (Supplementary Materials, Figure S1). Differential expression analysis of NRGs was performed using 23 COPD lung tissue samples and 9 normal lung tissue samples from GSE38974 (Figure 2A). The results identified 49 differentially expressed NRGs, including 32 upregulated (Figure 2B) and 17 downregulated genes (Figure 2C). Their differential expression patterns in COPD and normal lung tissues are shown in Figure S2 in the Supplementary Materials.

### 3.2. Enrichment Analysis of Differentially Expressed NRGs and Mechanism Exploration

**Figure 3:**
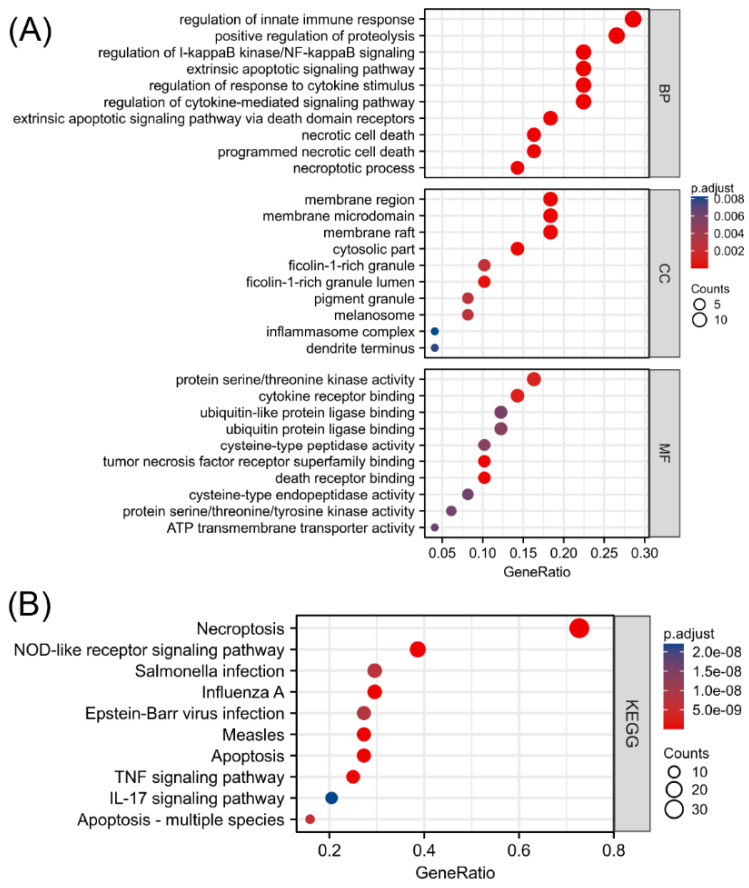
Functional enrichment analysis of differentially expressed NRGs. (**A**) Top 10 enriched biological processes, molecular functions, and cellular components identified by the GO analysis. (**B**) Top 10 significant signaling pathways identified by the KEGG analysis.

To explore the functional annotations of the 49 NRGs, GO and KEGG enrichment analyses were performed (Supplementary Materials, Tables S2 and S3). As shown in Figure 3A, programmed necrotic cell death, regulation of cytokine-mediated signaling pathway, extrinsic apoptotic signaling via death domain receptors, regulation of response to cytokine stimulus, and necrotic cell death were significantly enriched biological functions. The greatest number of NRGs were involved in regulating the innate immune response. Moreover, the KEGG enrichment analysis revealed that 49 biomarkers associated with necroptosis were significantly correlated with necroptosis, the NOD-like receptor signaling pathway, influenza A, apoptosis, and measles (Figure 3B). In addition to being correlated with cell death, the differentially expressed NRGs were mainly related to the regulatory functions of innate immune responses, regulation of I-kappaB kinase/NF-kappaB signaling, and immune-related pathways, such as the TNF signaling pathway and IL-17 signaling pathway. This finding suggested an interaction between NRGs and the immune system. The absolute value of the Z score represents the probability of regulation. The significantly enriched terms shown in Figure S3 in the Supplementary Materials are all likely to be positively regulated by the 49 differentially expressed NRGs.

### 3.3. PPI Network Construction and Correlation Analyses of Necroptosis-Related Hub Genes

**Figure 4:**
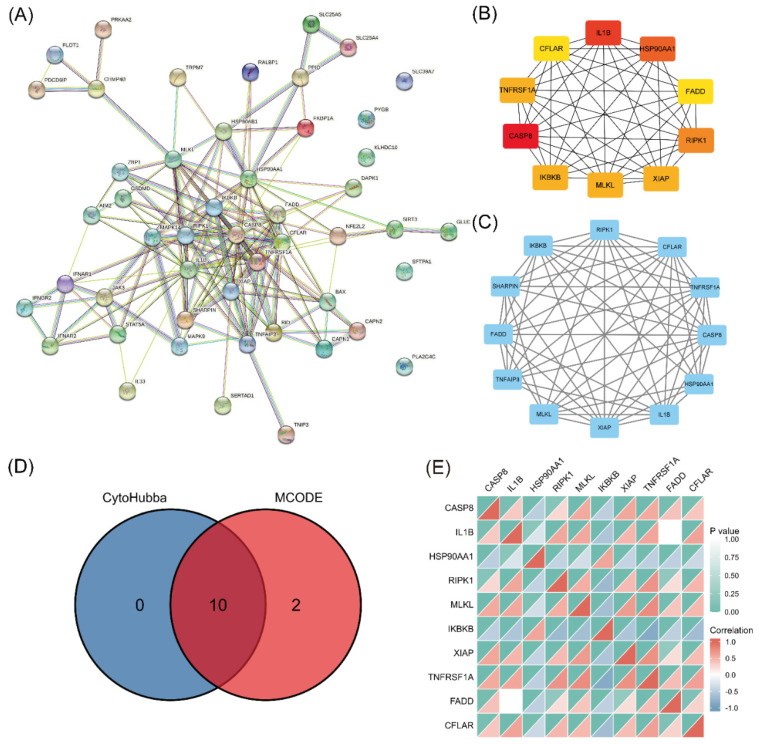
PPI analysis and hub gene identification. (**A**) PPI network of 49 differentially expressed NRGs constructed using STRING and Cytoscape. (**B**) Top 10 hub genes explored using cytoHubba. (**C**) Key module screened by MCODE clustering analysis. (**D**) Heatmap of the correlations of 10 necroptosis-related hub genes. (**E**) Overlap of predicted hub genes.

A PPI network was established using STRING and analyzed with Cytoscape (Figure 4A). The top 10 hub genes (CASP8, IL1B, HSP90AA1, RIPK1, MLKL, IKBKB, XIAP, TNFRSF1A, FADD, and CFLAR) were identified (Figure 4B). The key PPI module revealed a significant cluster composed of 12 nodes and 60 edges, including the genes RIPK3, CFLAR, TNFRSF1A, CASP8, HSP90AA1, IL1B, XIAP, MLKL, TNFAIP3, FADD, SHARPIN, and IKBKB (Figure 4C). The intersection of the results from the two analyses identified 10 genes as the final set of predicted hub genes related to necroptosis (Figure 4D). The heatmap revealed a certain degree of interaction among the expression levels of the 10 hub genes (Figure 4E).

### 3.4. Immune Infiltration Profiling

**Figure 5:**
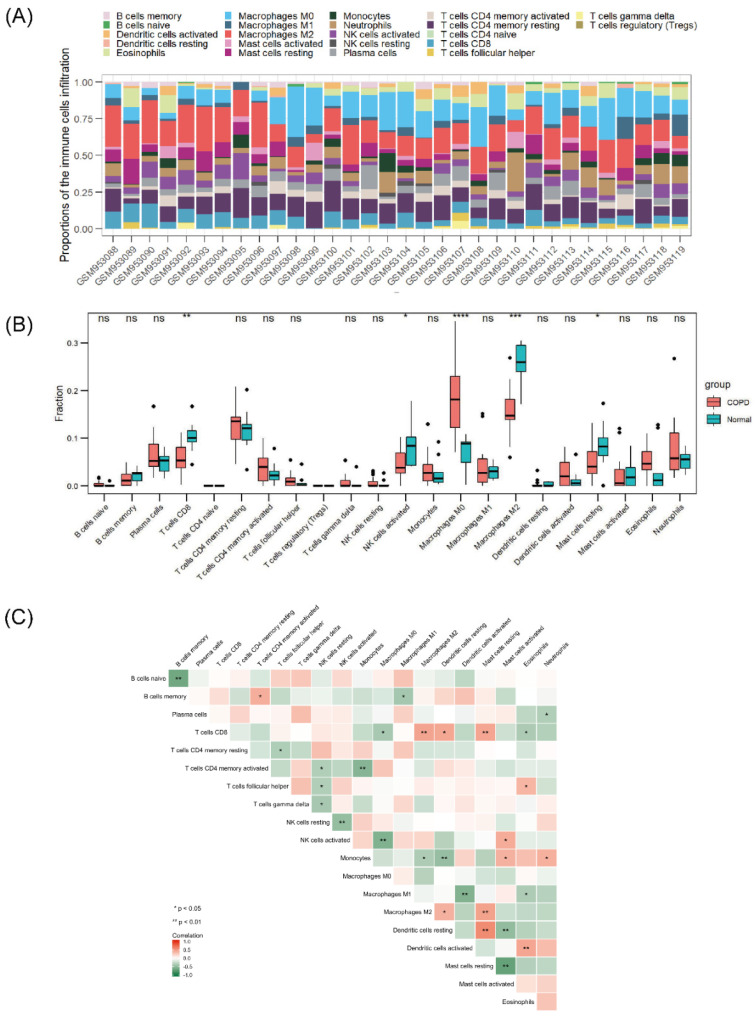
Landscape of immune infiltration in COPD. (**A**) Stack bar chart of immune cells. (**B**) Box plot of the immune cell proportions. (**C**) Heatmap of the correlation matrix of immune cells. ns *p* > 0.05, * *p* < 0.05, ** *p* < 0.01, *** *p* < 0.001, **** *p* < 0.0001.

**Figure 6:**
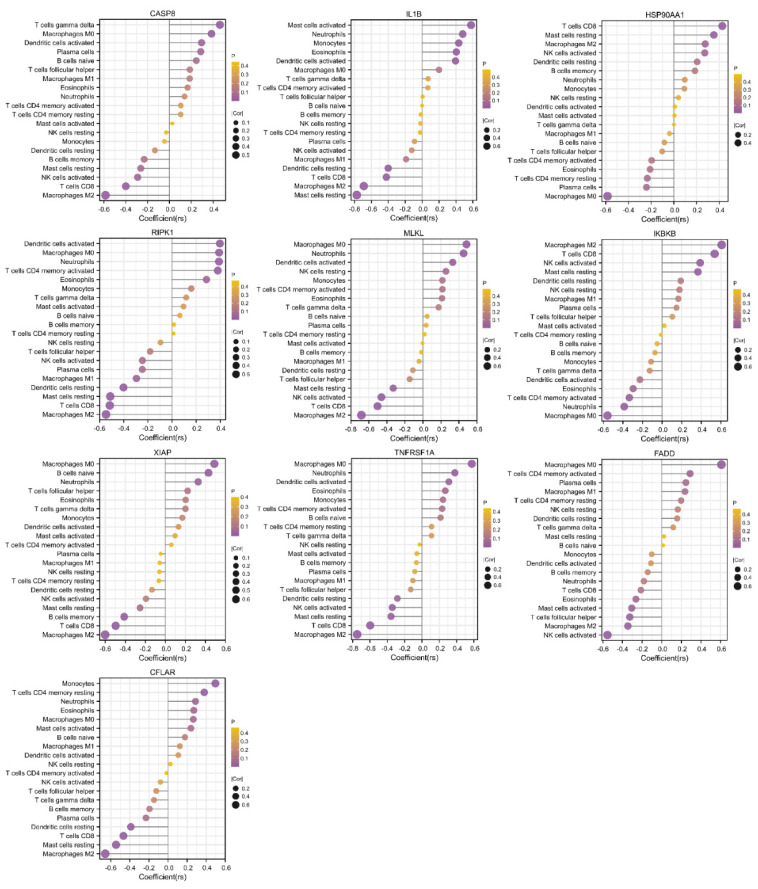
Correlations between hub genes and infiltrating immune cells. The size of the dot indicates the strength of the association between the gene and the immune cell type; a larger dot indicates a stronger correlation. The dot’s color represents the *p*-value; the more purple the color is, the smaller the *p*-value.

According to the aforementioned functional enrichment analysis, we found that NRGs in COPD seemed to show some connection with human immunity regulation. The GSE38974 dataset was selected for the analysis of immune cell infiltration. Figure 5A shows the general distribution of immune cells in individuals; specifically, M2 and M0 macrophages, resting memory CD4+ T cells, and CD8+ T cells accounted for the majority of all infiltrating immune cells. Compared with that in normal lung tissue, the infiltration of CD8+ T cells, activated natural killer (NK) cells, M2 macrophages, and resting mast cells in COPD lung tissue was relatively reduced, whereas that of M0 macrophages was enhanced (Figure 5B). The correlations among different infiltrating immune cells are shown in Figure 5C. CD8+ T cells, M2 macrophages, and resting mast cells were positively correlated, and CD8+ T cells were negatively correlated with M0 macrophages and eosinophils. Activated NK cells were negatively correlated with M0 macrophages, M2 macrophages, and monocytes, whereas resting dendritic cells were positively correlated with CD8+ cells, M2 macrophages, and resting mast cells. The correlations between necroptosis-related hub genes and infiltrating immune cells are shown in Figure 6. Most of the hub genes were positively correlated with M0 macrophage infiltration but inversely correlated with M2 macrophage and CD8+ T-cell infiltration.

### 3.5. Validation of Necroptosis-Related Hub Genes and Immune Infiltration

**Figure 7:**
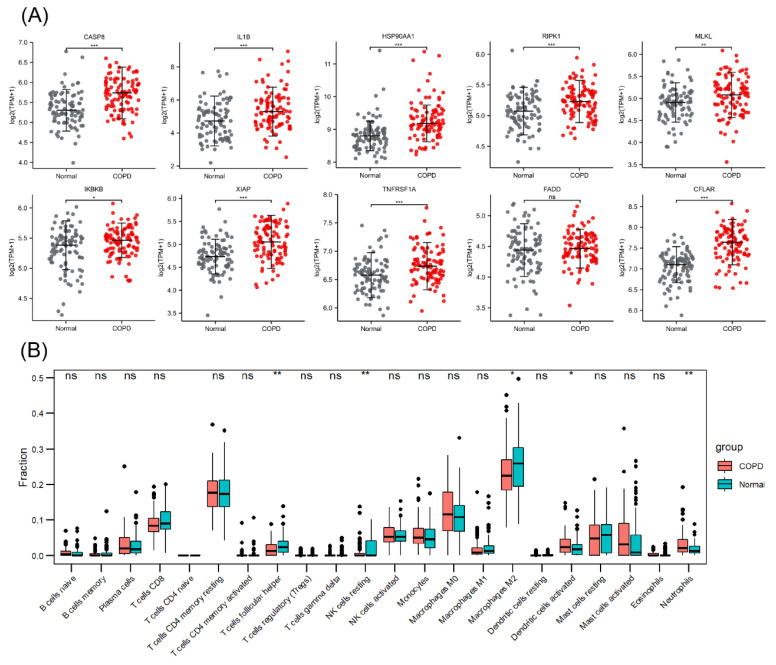
Validation of necroptosis-related hub genes and immune infiltration in an external dataset. (**A**) Expression of 10 necroptosis-related hub genes. (**B**) Box plot of the immune cell proportions. ns *p* > 0.05, * *p* < 0.05, ** *p* < 0.01, *** *p* < 0.001.

The PCA plot of the high-throughput sequencing dataset GSE57148 is shown in Figure S4 in the Supplementary Materials. The GSE57148 dataset was used to verify the expression levels of the abovementioned 10 necroptosis-related hub genes (Figure 7A). Among these genes, HSP90AA1 and IKBKB exhibited expression trends opposite to those in GSE38974, and no significant difference in FADD expression was detected. The reason for this finding may be sample heterogeneity. Figure 7B shows the immune infiltration information, indicating that M2 macrophage and resting NK-cell infiltration was attenuated in COPD samples and that neutrophil infiltration was enhanced.

**Figure 8:**
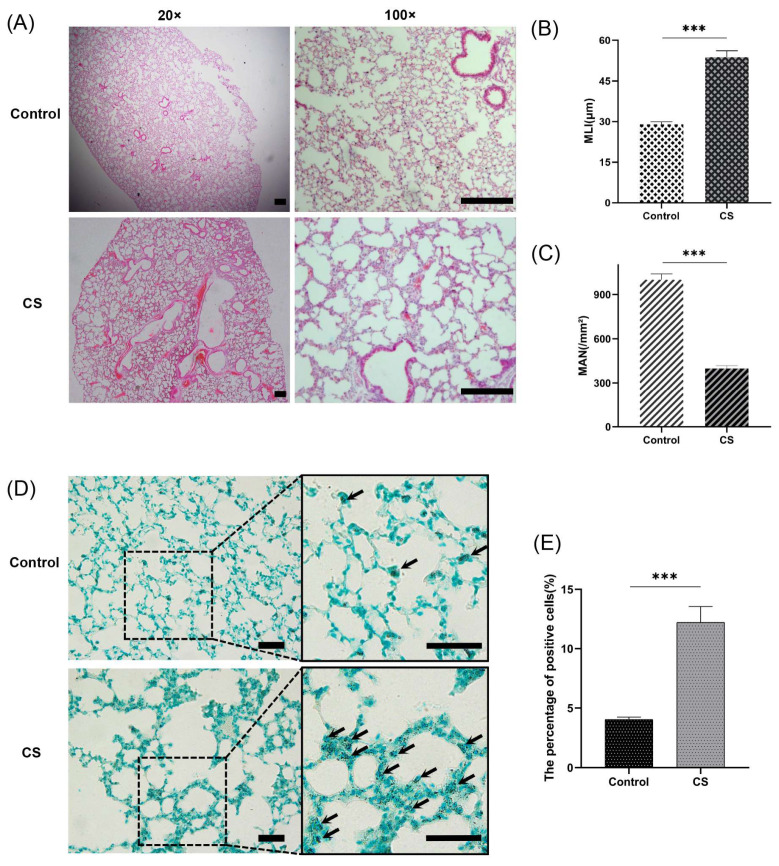
A CS-induced emphysema mouse model was established successfully, and cell death was significantly enhanced in lung tissues from mice in the CS group. (**A**) Photomicrograph of H&E-stained slides of tissues from mice in the normal control and CS groups. Representative images of micrographs at 20× and 100× magnification. Scale bar = 200 μm. (**B**) Morphometric analysis of the MLI (*n* = 6). (**C**) Morphometric analysis of the MAN (*n* = 6). (**D**) Photomicrograph of TUNEL-stained slides (200× magnification). Scale bar = 50 μm. (**E**) Assessment of TUNEL-positive cells. *** *p* < 0.001.

**Figure 9:**
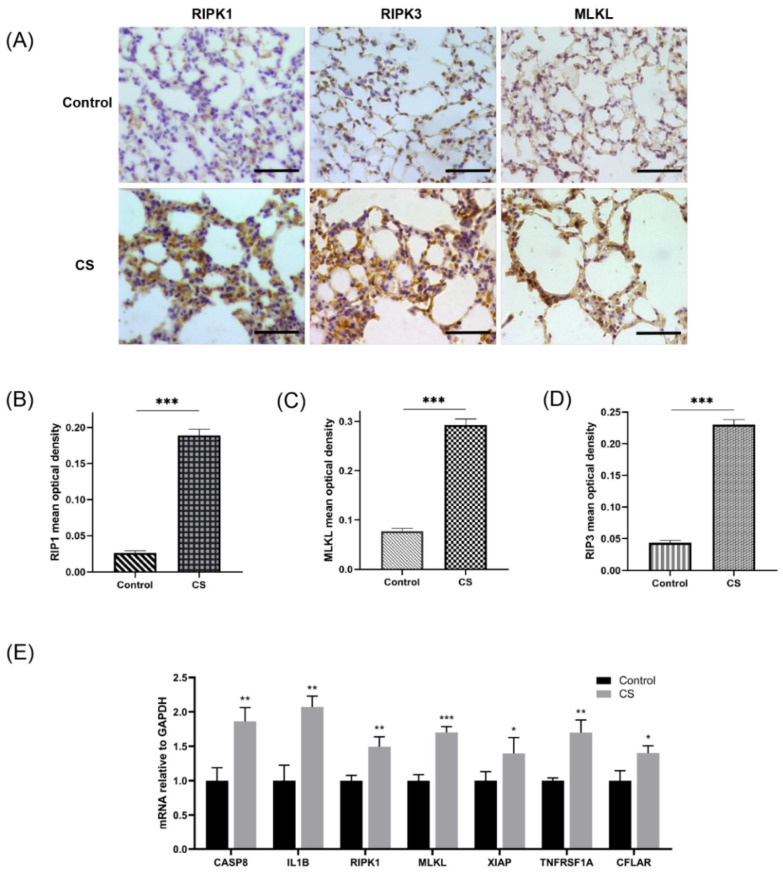
Assessment of necroptosis in lung tissues of experimental mice. (**A**) Photomicrograph of slides of tissues from mice in the normal control group and CS group immunohistochemically stained for RIPK1, RIPK3, and MLKL (400× magnification). Scale bar = 50 μm. (**B**) Quantification of RIPK1 (*n* = 6). (**C**) Quantification of RIPK3 (*n* = 6). (**D**) Quantification of MLKL (*n* = 6). (**E**) Relative mRNA expression levels of necroptosis-related hub genes in lung tissues (*n* = 4). * *p* < 0.05, ** *p* < 0.01, *** *p* < 0.001.

An emphysema model was established by subjecting the mice in the CS group to whole-body smoke exposure for 12 weeks. H&E staining of mouse lung tissues showed that the CS group exhibited a significantly decreased MAN and an increased MLI compared with the normal control group, which indicated that the emphysema animal model was successfully established (Figure 8A–C). Previous studies have confirmed that CS can induce necroptosis of lung tissue in COPD and experimental emphysema [14,15,16]. We performed TUNEL staining to assess cell death and found an increase in the number of positive cells in the lungs of the mice in the CS group (Figure 8D,E). Immunohistochemical staining analysis showed that the expression levels of RIPK1, RIPK3, and MLKL in alveolar epithelial cells in the CS group were higher than those in the normal control group (Figure 9A–D), indicating that necroptosis was enhanced in the lungs of mice with emphysema. The mRNA expression levels of 7 NRGs, CASP8, IL1B, RIPK1, MLKL, XIAP, TNFRSF1A, and CFLAR, were verified again by qRT–PCR (Figure 9E). The results were very similar to those obtained from the bioinformatics analysis. These results further suggest that CASP8, IL1B, RIPK1, MLKL, XIAP, TNFRSF1A, and CFLAR are potential biomarkers of necroptosis in COPD.

### 3.6. Diagnostic Value of Necroptosis-Related Hub Genes in COPD

**Figure 10:**
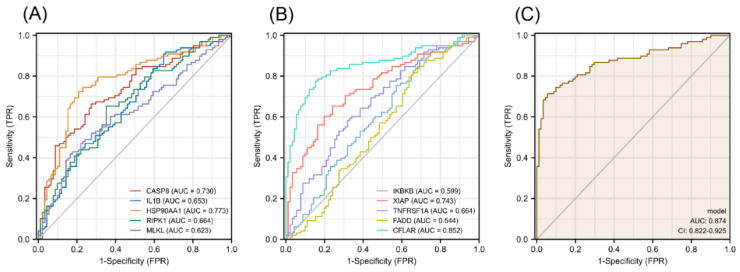
Diagnostic efficacy of necroptosis-related hub genes for COPD in the external dataset GSE57148. (**A**,**B**) ROC curves estimating the diagnostic performance of each characteristic gene. (**C**) ROC curve analysis by combining the expression levels of 7 validated necroptosis-related hub genes.

The diagnostic efficacy of necroptosis-related hub genes for COPD in GSE38974 is shown in Figure S5 in the Supplementary Materials, and all the candidate genes possess a high diagnostic value. The GSE57148 dataset was used to validate the diagnostic efficacy of the biomarkers for COPD. The diagnostic values of these genes were as follows (all greater than 0.5): CASP8, AUC = 0.730; IL1B, AUC = 0.653; HSP90AA1, AUC = 0.773; RIPK1, AUC = 0.664; MLKL, AUC = 0.623; IKBKB, AUC = 0.599; XIAP, AUC = 0.743; TNFRSF1A, AUC = 0.664; FADD, AUC = 0.544; and CFLAR, AUC = 0.852 (Figure 10A,B). Seven validated hub genes CASP8, IL1B, RIPK1, MLKL, XIAP, TNFRSF1A, and CFLAR were fitted into one variable, and the AUC was 0.874, demonstrating a favorable diagnostic performance in predicting COPD (Figure 10C).

### 3.7. TF–miRNA Coregulatory Network

**Figure 11:**
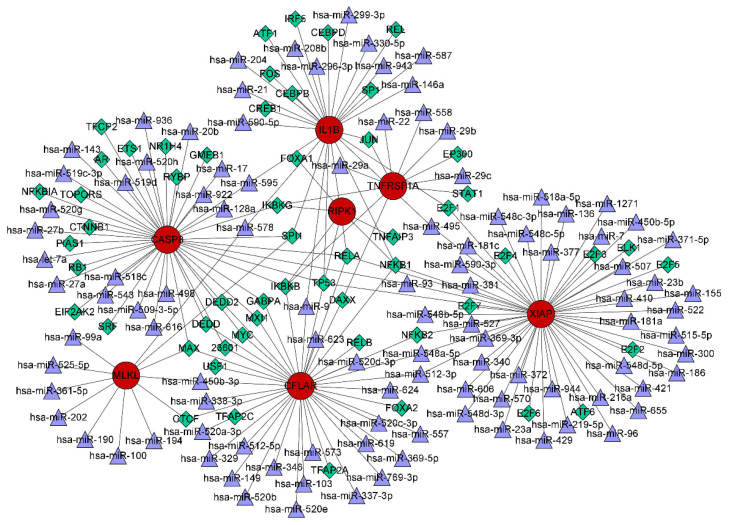
TF–miRNA coregulatory network consisting of 168 nodes and 199 edges and involving 56 TFs and 105 miRNAs. The nodes in red are the 7 confirmed necroptosis-related hub genes, the purple triangles represent miRNAs, and the green diamonds indicate TFs.

The interactions of TFs and miRNAs with the 7 confirmed NRGs are depicted in a TF–miRNA coregulatory network (Figure 11), which may provide clues to the regulation of NRG expression. The coregulatory network consists of 168 nodes and 199 edges and involves 56 TFs and 105 miRNAs.

### 3.8. Molecular Identification of Candidate Drugs

**Figure 12:**
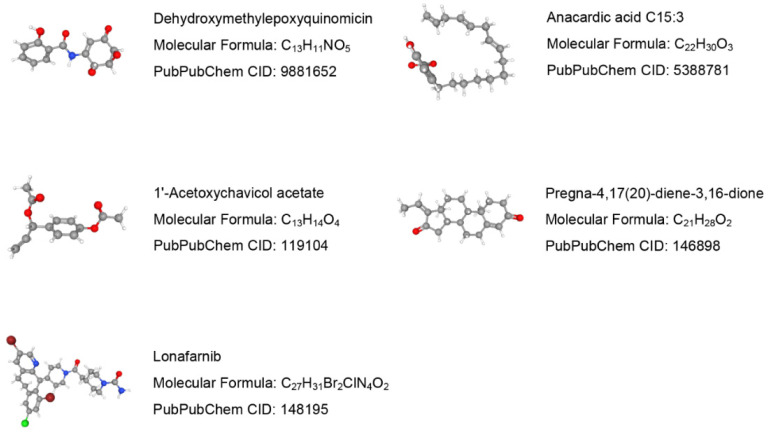
Prediction results of potential small molecule drugs for treating COPD based on 7 target genes.

Drug molecules targeting the 7 validated NRGs were searched in the DSigDB database. The top 10 predicted potential drugs according to the combined scores are shown in Table S3 in the Supplementary Materials. The molecular structures of the top 5 candidate drugs, dehydroxymethylepoxyquinomicin (DHMEQ), anacardic acid, 1′-acetoxychavicol acetate, pregna-4,17(20)-diene-3,16-dione, and lonafarnib, were retrieved from the PubChem database (Figure 12).

## 4. Discussion

Necroptosis has been implicated in the pathogenesis of several human pulmonary diseases, such as acute respiratory distress syndrome, COVID-19, asthma, and idiopathic pulmonary fibrosis [45]. However, COPD is a heterogeneous and complex progressive inflammatory disease, and studies on the role and molecular mechanisms of necroptosis in COPD are just beginning. This study constitutes the first bioinformatics analysis of NRGs in the pathogenesis of human COPD.

In this study, we first obtained 49 differentially expressed NRGs (32 upregulated and 17 downregulated genes) by analyzing the gene expression profiles of lung tissues from COPD patients and normal controls, which indicated that NRGs are indeed involved in the pathogenesis and progression of COPD. To understand the function and role of the differentially expressed NRGs, we performed enrichment analyses. We found that the biological process mainly involved programmed necrotic cell death, regulation of cytokine-mediated signaling pathway, extrinsic apoptotic signaling pathway via death domain receptors, regulation of response to cytokine stimulus, and necrotic cell death. Abnormal cell death is closely related to the development of emphysema in COPD [14,46,47,48,49] and involves various forms, such as apoptosis, necrosis, and programmed cell necrosis. Apoptosis was once considered the only regulated cell death mechanism, and increased numbers of apoptotic alveolar, bronchiolar, and endothelial cells have been observed in lung tissue from patients with COPD [50,51]. Necroptosis is a form of genetically encoded necrotic cell death involving rupture of the plasma membrane and therefore is a strong inducer of inflammation. It has been demonstrated that the release of damage-related molecular patterns (DAMPs) from CS-induced necroptosis triggers the production of proinflammatory cytokines [52]. Furthermore, a GO enrichment analysis indicated that several NRGs were involved in regulating innate immune response. A KEGG enrichment analysis revealed that differentially expressed NRGs were primarily engaged in inflammatory immune response pathways, including the NOD-like receptor signaling pathway, tumor necrosis factor (TNF) signaling pathway, and IL-17 signaling pathway. Ample evidence shows that TNF can induce necroptosis [53,54], and several necroptosis effectors have been reported to engage in crosstalk with the NLRP3 inflammasome to induce its activation [55,56,57]. A recent study found that theaflavin-3,3′-digallate attenuated emphysema in mice by suppressing necroptosis and significantly decreased the TNF-α and IL-1β levels [58]. Interleukin 17 (IL-17), mainly secreted by T-helper (Th) 17 cells, plays a vital role in autoimmune diseases. Jing Xiong et al. proposed that the B lymphocyte RANKL pathway is involved in IL-17A-dependent lymphoid neogenesis in COPD [59]. A recent study also confirmed that airway epithelium-derived IL-17A can amplify inflammation and increase mucus production in COPD pathogenesis in an autocrine manner [60]. These findings provide new insights into the function of necroptosis in COPD.

Adaptive and innate immune responses to risk factors contribute to the immunopathology of COPD [61]. We calculated the abundance of 22 immune cells based on the microarray profiles of normal and COPD lung tissue samples and obtained a comprehensive view of the immune infiltration status. In our study, a significant increase in infiltrating macrophages was found in COPD lung tissues, and a significant increase in the M0 macrophage numbers and a significant decrease in the M2 macrophage numbers were observed. This finding contradicts those reported by Erica Bazzan [62]. We hypothesize that the increase in M0 macrophages in COPD represents an enhanced reserve capacity in preparation for further polarization and that the decrease in the number of M2 macrophages implies that the anti-inflammatory and repair capacity is weakened in COPD. We also found that CD8+ T lymphocyte infiltration was reduced in COPD but not significantly altered in COPD lung tissues in the validation dataset GSE57148. Interestingly, regarding CD8+ T-cell expression in COPD, previous studies have not yielded consistent conclusions. Eapen et al. observed fewer CD8+ T cells in the large airways of smokers with and without COPD [63]. Forsslund et al. also reported a lower percentage of CD8+ T lymphocytes in the peripheral blood of smokers with and without COPD [64]. However, other reports have mentioned increased quantities of CD8+ T lymphocytes in COPD [65,66,67]. It has been demonstrated that the heterogeneity of macrophages and the activation of T cells are dependent on external stimuli and the microenvironment [68]. Several factors, including smoking status, severity of COPD, acute exacerbations, and corticosteroid use, may contribute to different conclusions [69,70,71]. Additionally, in the validation dataset, the degree of neutrophil infiltration was enhanced in lung tissue samples from COPD patients, which has been extensively demonstrated in previous studies [72,73].

The PPI analysis demonstrated that the proteins encoded by the 49 differentially expressed NRGs interacted and identified 10 necroptosis-related hub genes, including CASP8, IL1B, HSP90AA1, RIPK1, MLKL, IKBKB, XIAP, TNFRSF1A, FADD, and CFLAR. Previous studies have reported that some of these hub genes affect immune responses by regulating necroptosis. For example, XIAP mediates TNFα-induced neutrophil necroptosis [74], and CFLAR plays a critical role in autophagy, necroptosis, and apoptosis in T lymphocytes [75]. Our study demonstrated substantial correlations between necroptosis and immune cell infiltration in COPD, and some were consistent with those identified in previous studies. Notably, RIPK1, MLKL, XIAP, and CFLAR were significantly positively correlated with M0 macrophages but negatively correlated with M2 macrophages. Neutrophil infiltration was positively correlated with RIPK1, MLKL, and XIAP, and monocyte infiltration was positively correlated with CFLAR. These results imply that necroptosis drives the development of COPD by regulating the immune response, which provides a hypothesis and basis for further research. We also evaluated and validated the diagnostic efficacy of these 10 hub genes in the external dataset, and their AUCs were all greater than 0.5. Among these genes, CFLAR, which had an AUC greater than 0.80, is considered to have the best capability to diagnose COPD with excellent specificity and sensitivity. The combination of the 7 validated hub genes showed better discrimination (AUC = 0.874) than each gene alone.

TFs are modular proteins that regulate gene transcription by binding with target genes, and miRNAs can silence target gene expression through mRNA degradation or translational inhibition [76]. TFs and miRNAs can jointly regulate common target gene expression and play critical roles in multiple biological processes. The necroptosis-related hub genes were validated using an independent external dataset and animal experiments to achieve improved accuracy, and 7 genes were ultimately screened. A TF–miRNA coregulatory network was constructed to explore the upstream regulatory biomolecules of the 7 necroptosis-related hub genes, and 105 miRNAs and 56 TFs were identified. Among the most interactive TFs, TP53 exhibited the highest degree value of 5. A previous study confirmed that sirtuin 3-induced necroptosis in small-cell lung cancer is associated with the expression of mutant p53 [77]. Furthermore, the current study highlights the predicted potential drugs targeting the 7 validated necroptosis-related hub genes. DHMEQ, a novel NF-kappaB inhibitor, may exert therapeutic effects on allergic inflammation and airway remodeling in asthmatic mice [78]. However, the application of DHMEQ in COPD treatment has not yet been studied. The therapeutic effects of 1′-acetoxychavicol acetate on pulmonary inflammatory diseases also suggest that this drug may be useful for COPD [79,80]. The predicted potential drugs should be considered for further verification by chemical experiments to shed light on new therapeutic strategies for preventing COPD progression.

This study has some limitations. First, we performed only a preliminary exploration of the correlation between NRG expression and immune cell infiltration. The complex regulatory mechanisms and interactions between necroptosis and immune cell infiltration have not been specifically studied in depth. Second, quite a few samples are included in GSE38974, and the datasets used in this study do not provide extensive information on clinical characteristics or prognostic information. Although the GSE38974 dataset has been used alone in many published studies [24,81,82,83], the results may be subject to minor errors. To facilitate our future research on COPD, we are collecting our own clinical samples and relevant clinical information. Third, lung macrophages remain poorly understood in COPD. We only investigated macrophage polarization according to M1 and M2 classifications in the study. However, based on the available evidence, changes in M1 and M2 macrophages did not yield consistent conclusions, supporting the idea that classical M1 or M2 phenotypes are insufficient to explain lung macrophage differentiation and dyshomeostasis in COPD. A comprehensive study of macrophage and monocyte immunophenotyping will be needed to develop more accurate biomarkers in the future. Moreover, we only predicted the coregulatory networks of upstream TFs and miRNAs as well as potential therapeutic agents. These predictions were based on bioinformatics methods, and subsequent in vivo and in vitro confirmatory experiments are thus needed.

## 5. Conclusions

Bioinformatics analyses of necroptosis in COPD have rarely been reported. Our analysis revealed that the expression levels of NRGs significantly differed between COPD and normal lung tissues. Moreover, this study not only obtained insights into the landscape of immune cells associated with COPD and their correlation with NRGs but also identified effective diagnostic biomarkers for COPD. Furthermore, we validated 7 necroptosis-related hub genes of COPD. The interactions of TF and miRNA with their common NRGs and potential new therapeutic drugs for medical interventions were then identified.

## Figures and Tables

**Table 1:** Characteristics of the datasets used in this study.

Accession	Platform	Sample Type	Total	Normal	COPD	Gene	Contributors
GSE38974	GPL4133	Lung tissue	32	9	23	mRNA	Ezzie M.E. et al.
GSE57148	GPL11154	Lung tissue	189	91	98	mRNA	Kim W.J. et al.

## Data Availability

The data used to support the findings of this study are included within the article.

## Supplementary Materials

The following supporting information can be downloaded at: https://www.mdpi.com/article/10.3390/biom13030482/s1, Table S1: Necroptosis gene set from databases; Table S2: Gene Ontology enrichment analysis; Table S3: Kyoto Encyclopedia of Genes and Genomes pathway analysis; Table S4: PCR primers; Table S5: Predicted potential drugs from the DSigDB database. Figure S1: PCA plot of samples in GSE38974; Figure S2: Heatmap of 49 differentially expressed NRGs; Figure S3: Donut plot of significant terms combined with logFC values; Figure S4: PCA plot of samples in GSE57148; Figure S5: Diagnostic efficacy of necroptosis-related hub genes in the GSE38974 dataset.
