# Supplementary material for: Identification and Analysis of Necroptosis-Related Genes in COPD by Bioinformatics and Experimental Verification
Source: Biomolecules. 2023 Mar 6;13(3):482. doi: 10.3390/biom13030482 (PMC10046193; doi:10.3390/biom13030482)
Supplement: Supplementary file 1 [file biomolecules-13-00482-s001.zip › Figure S3.pdf]

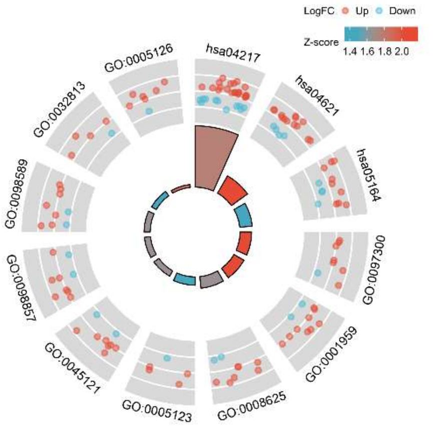

Supplementary Materials Figure S3. Donut plot of significant terms combined with logFC values. The height of the inner circle column represents the adjusted P value, with a higher height indicating a smaller P value. The fill color represents the Z score of each term.
