# Supplementary material for: Identification and Analysis of Necroptosis-Related Genes in COPD by Bioinformatics and Experimental Verification
Source: Biomolecules. 2023 Mar 6;13(3):482. doi: 10.3390/biom13030482 (PMC10046193; doi:10.3390/biom13030482)
Supplement: Supplementary file 1 [file biomolecules-13-00482-s001.zip › Table S1.pdf]

Supplementary Materials Table S1. Necroptosis gene set from databases.

Gene Symbol	Description
TNF	tumor necrosis factor
TNFRSF1A	TNF receptor superfamily member 1A
TRADD	TNFRSF1A associated via death domain
TRAF2	TNF receptor associated factor 2
TRAF5	TNF receptor associated factor 5
RIPK1	receptor interacting serine/threonine kinase 1
BIRC2	baculoviral IAP repeat containing 2
BIRC3	baculoviral IAP repeat containing 3
XIAP	X-linked inhibitor of apoptosis
RBCK1	RANBP2-type and C3HC4-type zinc finger containing 1
RNF31	ring finger protein 31
SHARPIN	SHANK associated RH domain interactor
SPATA2L	spermatogenesis associated 2 like
SPATA2	spermatogenesis associated 2
CYLD	CYLD lysine 63 deubiquitinase
FADD	Fas associated via death domain
CASP8	caspase 8
CFLAR	CASP8 and FADD like apoptosis regulator
RIPK3	receptor interacting serine/threonine kinase 3

CYBB	cytochrome b-245 beta chain
CAMK2A	calcium/calmodulin dependent protein kinase II alpha
CAMK2D	calcium/calmodulin dependent protein kinase II delta
CAMK2B	calcium/calmodulin dependent protein kinase II beta
CAMK2G	calcium/calmodulin dependent protein kinase II gamma
SLC25A4	solute carrier family 25 member 4
SLC25A5	solute carrier family 25 member 5
SLC25A6	solute carrier family 25 member 6
SLC25A31	solute carrier family 25 member 31
PPID	peptidylprolyl isomerase D
VDAC1	voltage dependent anion channel 1
VDAC2	voltage dependent anion channel 2
VDAC3	voltage dependent anion channel 3
GLUD2	glutamate dehydrogenase 2
GLUD1	glutamate dehydrogenase 1
GLUL	glutamate-ammonia ligase
PYGL	glycogen phosphorylase L
PYGM	glycogen phosphorylase, muscle associated
PYGB	glycogen phosphorylase B
MAPK8	mitogen-activated protein kinase 8

MAPK10	mitogen-activated protein kinase 10
MAPK9	mitogen-activated protein kinase 9
FTH1	ferritin heavy chain 1
FTL	ferritin light chain
PLA2G4E	phospholipase A2 group IVE
PLA2G4A	phospholipase A2 group IVA
JMJD7-PLA2G4B	JMJD7-PLA2G4B readthrough
PLA2G4B	phospholipase A2 group IVB
PLA2G4C	phospholipase A2 group IVC
PLA2G4D	phospholipase A2 group IVD
PLA2G4F	phospholipase A2 group IVF
ALOX15	arachidonate 15-lipoxygenase
CAPN1	calpain 1
CAPN2	calpain 2
SMPD1	sphingomyelin phosphodiesterase 1
MLKL	mixed lineage kinase domain like pseudokinase
PGAM5	PGAM family member 5, mitochondrial serine/threonine protein phosphatase
DNM1L	dynamin 1 like
NLRP3	NLR family pyrin domain containing 3
PYCARD	PYD and CARD domain containing

CASP1	caspase 1
IL1B	interleukin 1 beta
CHMP2A	charged multivesicular body protein 2A
CHMP2B	charged multivesicular body protein 2B
CHMP3	charged multivesicular body protein 3
RNF103-CHMP3	RNF103-CHMP3 readthrough
CHMP4B	charged multivesicular body protein 4B
CHMP4A	charged multivesicular body protein 4A
CHMP4C	charged multivesicular body protein 4C
CHMP6	charged multivesicular body protein 6
VPS4B	vacuolar protein sorting 4 homolog B
VPS4A	vacuolar protein sorting 4 homolog A
CHMP1B	charged multivesicular body protein 1B
CHMP1A	charged multivesicular body protein 1A
CHMP5	charged multivesicular body protein 5
CHMP7	charged multivesicular body protein 7
TRPM7	transient receptor potential cation channel subfamily M member 7
IL1A	interleukin 1 alpha
IL33	interleukin 33
HMGB1	high mobility group box 1

TNFSF10	TNF superfamily member 10
TNFRSF10A	TNF receptor superfamily member 10a
TNFRSF10B	TNF receptor superfamily member 10b
FASLG	Fas ligand
FAS	Fas cell surface death receptor
FAF1	Fas associated factor 1
IFNA1	interferon alpha 1
IFNA2	interferon alpha 2
IFNA4	interferon alpha 4
IFNA5	interferon alpha 5
IFNA6	interferon alpha 6
IFNA7	interferon alpha 7
IFNA8	interferon alpha 8
IFNA10	interferon alpha 10
IFNA13	interferon alpha 13
IFNA14	interferon alpha 14
IFNA16	interferon alpha 16
IFNA17	interferon alpha 17
IFNA21	interferon alpha 21
IFNB1	interferon beta 1

IFNG	interferon gamma
IFNAR1	interferon alpha and beta receptor subunit 1
IFNAR2	interferon alpha and beta receptor subunit 2
IFNGR1	interferon gamma receptor 1
IFNGR2	interferon gamma receptor 2
JAK1	Janus kinase 1
JAK2	Janus kinase 2
JAK3	Janus kinase 3
TYK2	tyrosine kinase 2
STAT1	signal transducer and activator of transcription 1
STAT2	signal transducer and activator of transcription 2
STAT3	signal transducer and activator of transcription 3
STAT4	signal transducer and activator of transcription 4
STAT5A	signal transducer and activator of transcription 5A
STAT5B	signal transducer and activator of transcription 5B
STAT6	signal transducer and activator of transcription 6
IRF9	interferon regulatory factor 9
EIF2AK2	eukaryotic translation initiation factor 2 alpha kinase 2
TLR4	toll like receptor 4
TICAM2	toll like receptor adaptor molecule 2

TICAM1	toll like receptor adaptor molecule 1
TLR3	toll like receptor 3
ZBP1	Z-DNA binding protein 1
USP21	ubiquitin specific peptidase 21
SQSTM1	sequestosome 1
HSP90AA1	heat shock protein 90 alpha family class A member 1
HSP90AB1	heat shock protein 90 alpha family class B member 1
TNFAIP3	TNF alpha induced protein 3
PARP1	poly(ADP-ribose) polymerase 1
BID	BH3 interacting domain death agonist
BAX	BCL2 associated X, apoptosis regulator
AIFM1	apoptosis inducing factor mitochondria associated 1
H2AX	H2A.X variant histone
H2AC20	H2A clustered histone 20
H2AC12	H2A clustered histone 12
H2AC1	H2A clustered histone 1
H2AW	H2A.W histone
H2AB3	H2A.B variant histone 3
H2AC8	H2A clustered histone 8
H2AC4	H2A clustered histone 4

MACROH2A2	macroH2A.2 histone
MACROH2A1	macroH2A.1 histone
H2AC19	H2A clustered histone 19
H2AJ	H2A.J histone
H2AB1	H2A.B variant histone 1
H2AC17	H2A clustered histone 17
H2AC18	H2A clustered histone 18
H2AC11	H2A clustered histone 11
H2AC21	H2A clustered histone 21
H2AZ2	H2A.Z variant histone 2
H2AC7	H2A clustered histone 7
H2AZ1	H2A.Z variant histone 1
H2AC15	H2A clustered histone 15
H2AC6	H2A clustered histone 6
H2AC13	H2A clustered histone 13
H2AC14	H2A clustered histone 14
H2AC16	H2A clustered histone 16
H2AB2	H2A.B variant histone 2
PPIA	peptidylprolyl isomerase A
BCL2	BCL2 apoptosis regulator

ITPK1	Inositol-Tetrakisphosphate 1-Kinase
IPMK	Inositol Polyphosphate Multikinase
MAP3K7	Mitogen-Activated Protein Kinase Kinase Kinase 7
CASP6	Caspase 6
PELI1	Pellino E3 Ubiquitin Protein Ligase 1
PGLYRP1	Peptidoglycan Recognition Protein 1
SIRT3	Sirtuin 3
TP53	Tumor Protein P53
MEFV	MEFV Innate Immuity Regulator, Pyrin
AIM2	Absent In Melanoma 2
UCHL1	Ubiquitin C-Terminal Hydrolase L1
STING1	Stimulator Of Interferon Response CGAMP Interactor 1
TNIP1	TNFAIP3 Interacting Protein 1
SERTAD1	SERTA Domain Containing 1
NFKB1	Nuclear Factor Kappa B Subunit 1
MAPK14	Mitogen-Activated Protein Kinase 14
KLHDC10	Kelch Domain Containing 10
SFTPA1	Surfactant Protein A1
GSK3B	Glycogen Synthase Kinase 3 Beta
MYC	MYC Proto-Oncogene, BHLH Transcription Factor

TNIP3	TNFAIP3 Interacting Protein 3
GJB1	Gap Junction Protein Beta 1
PTGES3	Prostaglandin E Synthase 3
UVRAG	UV Radiation Resistance Associated
BRD4	Bromodomain Containing 4
RB1	RB Transcriptional Corepressor 1
SIRT2	Sirtuin 2
IKBKB	Inhibitor Of Nuclear Factor Kappa B Kinase Subunit Beta
DAPK1	Death Associated Protein Kinase 1
FKBP1A	FKBP Prolyl Isomerase 1A
MIR425	MicroRNA 425
EZH2	Enhancer Of Zeste 2 Polycomb Repressive Complex 2 Subunit
NFE2L2	NFE2 Like BZIP Transcription Factor 2
CD274	CD274 Molecule
CXCL5	C-X-C Motif Chemokine Ligand 5
MIR29B1	MicroRNA 29b-1
AXL	AXL Receptor Tyrosine Kinase
MERTK	MER Proto-Oncogene, Tyrosine Kinase
TYRO3	TYRO3 Protein Tyrosine Kinase
SIRT6	Sirtuin 6

NAT2	N-Acetyltransferase 2
SLC39A7	Solute Carrier Family 39 Member 7
USP22	Ubiquitin Specific Peptidase 22
PANX1	Pannexin 1
PDCD6IP	Programmed Cell Death 6 Interacting Protein
FLOT1	Flotillin 1
FLOT2	Flotillin 2
IL37	Interleukin 37
DIABLO	Diablo IAP-Binding Mitochondrial Protein
FASN	Fatty Acid Synthase
CDK9	Cyclin Dependent Kinase 9
TIMM50	Translocase Of Inner Mitochondrial Membrane 50
SLC25A37	Solute Carrier Family 25 Member 37
PPP1R3G	Protein Phosphatase 1 Regulatory Subunit 3G
MIR7-1	MicroRNA 7-1
NFKBIA	NFKB Inhibitor Alpha
AURKC	Aurora Kinase C
NGFR	Nerve Growth Factor Receptor
FMR1	Fragile X Messenger Ribonucleoprotein 1
GNLY	Granulysin

HTRA2	HtrA Serine Peptidase 2
HSPA5	Heat Shock Protein Family A (Hsp70) Member 5
PRKAA2	Protein Kinase AMP-Activated Catalytic Subunit Alpha 2
PRKAA1	Protein Kinase AMP-Activated Catalytic Subunit Alpha 1
PITPNA	Phosphatidylinositol Transfer Protein Alpha
METTL3	Methyltransferase 3, N6-Adenosine-Methyltransferase Complex Catalytic Subunit
FNDC4	Fibronectin Type III Domain Containing 4
FNDC5	Fibronectin Type III Domain Containing 5
TXN	Thioredoxin
RALBP1	RalA Binding Protein 1
TP53I3	Tumor Protein P53 Inducible Protein 3
GSDMD	Gasdermin D
PRKN	Parkin RBR E3 Ubiquitin Protein Ligase
