# Supplementary material for: Identification and Analysis of Necroptosis-Related Genes in COPD by Bioinformatics and Experimental Verification
Source: Biomolecules. 2023 Mar 6;13(3):482. doi: 10.3390/biom13030482 (PMC10046193; doi:10.3390/biom13030482)
Supplement: Supplementary file 1 [file biomolecules-13-00482-s001.zip › Table S2.pdf]

Supplementary Materials Table S2. Gene Ontology enrichment analysis.

Category	GO ID	GO pathways	P-value	Count	zscore
BP	GO:0097300	programmed necrotic cell death	5.13E-13	8	2.12132
	GO:0001959	regulation of cytokine-mediated signaling pathway	8.67E-13	11	2.110579
	GO:0008625	extrinsic apoptotic signaling pathway via death domain receptors	1.07E-12	9	1.666667
	GO:0060759	regulation of response to cytokine stimulus	1.89E-12	11	2.110579
	GO:0070265	necrotic cell death	3.75E-12	8	2.12132
	GO:0045088	regulation of innate immune response	6.06E-12	14	2.672612
	GO:0045862	positive regulation of proteolysis	6.4E-12	13	0.83205
	GO:0097191	extrinsic apoptotic signaling pathway	1.13E-11	11	1.507557
	GO:0070266	necroptotic process	1.95E-11	7	1.889822
	GO:0043122	regulation of I-kappaB kinase/NF-kappaB signaling	2.08E-11	11	2.110579
MF	GO:0005123	death receptor binding	7.96E-10	5	1.341641
	GO:0032813	tumor necrosis factor receptor superfamily binding	1.66E-07	5	1.341641
	GO:0005126	cytokine receptor binding	1.29E-05	7	1.889822
	GO:0004674	protein serine/threonine kinase activity	2.49E-05	8	0
	GO:0031625	ubiquitin protein ligase binding	0.000142	6	0.816497
	GO:0008234	cysteine-type peptidase activity	0.000151	5	1.341641
	GO:0044389	ubiquitin-like protein ligase binding	0.000197	6	0.816497
	GO:0004712	protein serine/threonine/tyrosine kinase activity	0.000228	3	-0.57735
	GO:0004197	cysteine-type endopeptidase activity	0.000296	4	1
	GO:0005347	ATP transmembrane transporter activity	0.000333	2	0
CC	GO:0045121	membrane raft	7.09E-08	9	1.666667
	GO:0098857	membrane microdomain	7.28E-08	9	1.666667
	GO:0098589	membrane region	1E-07	9	1.666667
	GO:0044445	cytosolic part	2.44E-06	7	1.889822
	GO:1904813	ficolin-1-rich granule lumen	1.39E-05	5	1.341641
	GO:0101002	ficolin-1-rich granule	9.4E-05	5	1.341641
	GO:0042470	melanosome	0.000139	4	1
	GO:0048770	pigment granule	0.000139	4	1
	GO:0044292	dendrite terminus	0.000464	2	0
	GO:0061702	inflammasome complex	0.00054	2	1.414214
