# Supplementary material for: Identification and Analysis of Necroptosis-Related Genes in COPD by Bioinformatics and Experimental Verification
Source: Biomolecules. 2023 Mar 6;13(3):482. doi: 10.3390/biom13030482 (PMC10046193; doi:10.3390/biom13030482)
Supplement: Supplementary file 1 [file biomolecules-13-00482-s001.zip › Table S3.pdf]

Supplementary Materials Table S3. Kyoto Encyclopedia of Genes and Genomes pathway analysis.

ID	Pathways	P-value	Count	zscore
hsa04217	Necroptosis	1.7E-46	32	1.767767
hsa04621	NOD-like receptor signaling pathway	1.73E-17	17	2.182821
hsa05164	Influenza A	3.2E-12	13	1.38675
hsa04210	Apoptosis	4.25E-12	12	1.154701
hsa05162	Measles	5.52E-12	12	1.732051
hsa04668	TNF signaling pathway	1.16E-11	11	2.110579
hsa04215	Apoptosis - multiple species	2.63E-10	7	1.133893
hsa05132	Salmonella infection	3.69E-10	13	1.941451
hsa05169	Epstein-Barr virus infection	4.52E-10	12	1.732051
hsa04657	IL-17 signaling pathway	1.35E-09	9	1
