# Supplementary material for: Identification and Analysis of Necroptosis-Related Genes in COPD by Bioinformatics and Experimental Verification
Source: Biomolecules. 2023 Mar 6;13(3):482. doi: 10.3390/biom13030482 (PMC10046193; doi:10.3390/biom13030482)
Supplement: Supplementary file 1 [file biomolecules-13-00482-s001.zip › Table S4.pdf]

Supplementary Materials Table S4. PCR primers.

Gene	Gene ID	Forward primer sequence (Tm)	Reverse primer sequence (Tm)
Casp8	12370	TGCTTGGACTACATCCCACAC (61.8℃)	GTTGCAGTCTAGGAAGTTGACC (60.6℃)
Il1b	16176	CTTCAGGCAGGCAGTATC (60.7℃)	CAGCAGGTTATCATCATCATC (61.4℃)
Ripk1	19766	GAAGACAGACCTAGACAGCGG (61.6℃)	CCAGTAGCTTCACCACTCGAC (62.1℃)
Mlkl	74568	AATTGTACTCTGGGAAATTGCCA (60.2℃)	TCTCCAAGATTCCGTCCACAG (61.2℃)
Xiap	11798	AGTTGTCATGCGGCAATAGATAG (60.5℃)	CTGTCAGGGGCAAAAGGATTT (60.5℃)
Tnfrsf1a	21937	GGGGATACATCCATCAGGGGT (62.6℃)	GCTCGGACAGTCACTCACC (62℃)
Cflar	12633	GCTCCAGAATGGGCGAAGTAA (62.1℃)	ACGGATGTGCGGAGGTAAAAA (62℃)
Gapdh	14433	CAGCCGCATCTTCTTGTGC (60.2℃)	GGTAACCAGGCGTCCGATA (60.2℃)
