# Supplementary material for: Identification and Analysis of Necroptosis-Related Genes in COPD by Bioinformatics and Experimental Verification
Source: Biomolecules. 2023 Mar 6;13(3):482. doi: 10.3390/biom13030482 (PMC10046193; doi:10.3390/biom13030482)
Supplement: Supplementary file 1 [file biomolecules-13-00482-s001.zip › Table S5.pdf]

Supplementary Materials Table S5. Predicted potential drugs from the DSigDB database.

Drug	P-value	Combined Score	Genes
Dehydroxymethylepoxyquinomicin CTD 00004091	7.50E-09	28039.46744	CASP8, XIAP, CFLAR
Anacardic acid C15:3 CTD 00003117	9.19E-11	26754.53917	CASP8, IL1B, XIAP, CFLAR
1'-Acetoxychavicol acetate CTD 00002113	1.65E-10	22211.16608	IL1B, XIAP, CFLAR, TNFRSF1A
Pregna-4,17(20)-diene-3,16-dione CTD 00001094	5.29E-10	15360.26957	CASP8, IL1B, XIAP, CFLAR
Lonafarnib CTD 00003616	6.80E-08	10747.0207	CASP8, XIAP, CFLAR
beta-Solamarine CTD 00001251	5.77E-06	10714.52566	CASP8, TNFRSF1A
Aclarubicin CTD 00007056	5.77E-06	10714.52566	CASP8, XIAP
2,6,9,9-tetramethylcycloundeca-2,6,10-trien-1-one CTD 00003763	8.57E-08	9748.001391	XIAP, CFLAR, TNFRSF1A
Evodiamine CTD 00002158	8.57E-08	9748.001391	IL1B, XIAP, CFLAR
Cordycepin CTD 00002433	6.92E-06	9496.988892	CASP8, XIAP
