# Supplementary figures and images for: Identification and Analysis of Necroptosis-Related Genes in COPD by Bioinformatics and Experimental Verification
Source: Biomolecules. 2023 Mar 6;13(3):482. doi: 10.3390/biom13030482 (PMC10046193; doi:10.3390/biom13030482)

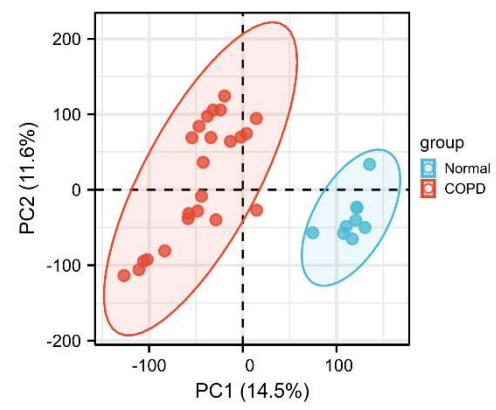

**Supplementary Materials Figure S1.** PCA plot of samples in GSE38974.

Supplement: Supplementary file 1 [file biomolecules-13-00482-s001.zip › Figure S1.pdf]

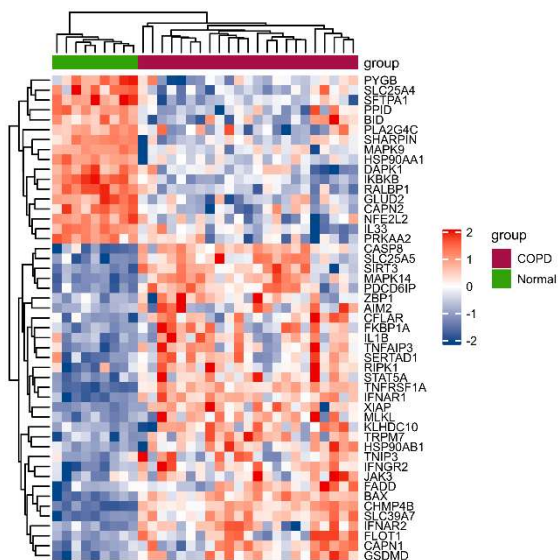

**Supplementary Materials Figure S2.** Heatmap of 49 differentially expressed NRGs.

Supplement: Supplementary file 1 [file biomolecules-13-00482-s001.zip › Figure S2.pdf]

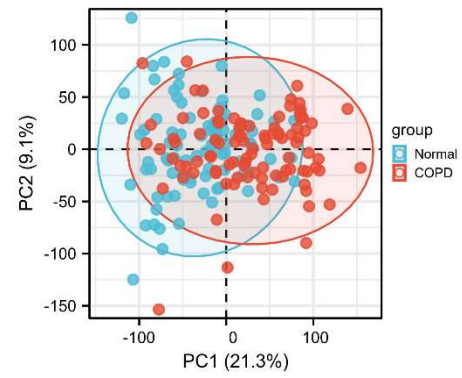

**Supplementary Materials Figure S4.** PCA plot of samples in GSE57148.

Supplement: Supplementary file 1 [file biomolecules-13-00482-s001.zip › Figure S4.pdf]
